# Supplementary material for: Changes in Neutrophil Count During Valganciclovir Therapy for Symptomatic Congenital Cytomegalovirus Infection
Source: Biomedicines. 2025 Jul 16;13(7):1739. doi: 10.3390/biomedicines13071739 (PMC12292696; doi:10.3390/biomedicines13071739)
Supplement: Supplementary file 1 [file biomedicines-13-01739-s001.zip › biomedicines-3728259-supplementary.pdf]

Supplemenatary Table S1. Exploration of patient factors associated with neutropenia occurring after two weeks of VGCV treatment (N of events: 8) using univariable logistic regression analyses

Characteristics	Odds ratio for neutropenia	95% confidence interval	P value
VGCV 32mg/kg (ref. 16mg/kg)	1.93	0.32 to 11.74	0.476
Gestational age (1 week increase)	0.71	0.51 to 0.97	0.034
Birthweight (100g increase)	0.87	0.75 to 1.02	0.092
Male (ref. female)	2.87	0.53 to 15.04	0.221
SGA (ref. absence)	1.13	0.21 to 5.97	0.890
Microcephaly (ref. absence)	1.70	0.31 to 9.37	0.542
Thrombocytopenia (ref. absence)	2.17	0.42 to 11.30	0.359
Liver disfunction (ref. absence)	0.76	0.12 to 4.75	0.771
Eye complications (ref. absence)	1.20	0.18 to 7.88	0.849
Brain imaging abnormalities (ref. absence)	0.67	0.05 to 8.52	0.755
ABR abnormality (ref. absence)	1.31	0.21 to 8.18	0.771
Blood CMV load before VGCV treatment (10 times increase)	1.15	0.51 to 2.63	0.734
Urine CMV load before VGCV treatment (10 times increase)	1.14	0.61 to 2.14	0.683

Abbreviations: SGA, small for gestational age; ABR, auditory brain stem response; CMV, congenital cytomegalovirus; VGCV, valganciclovir
